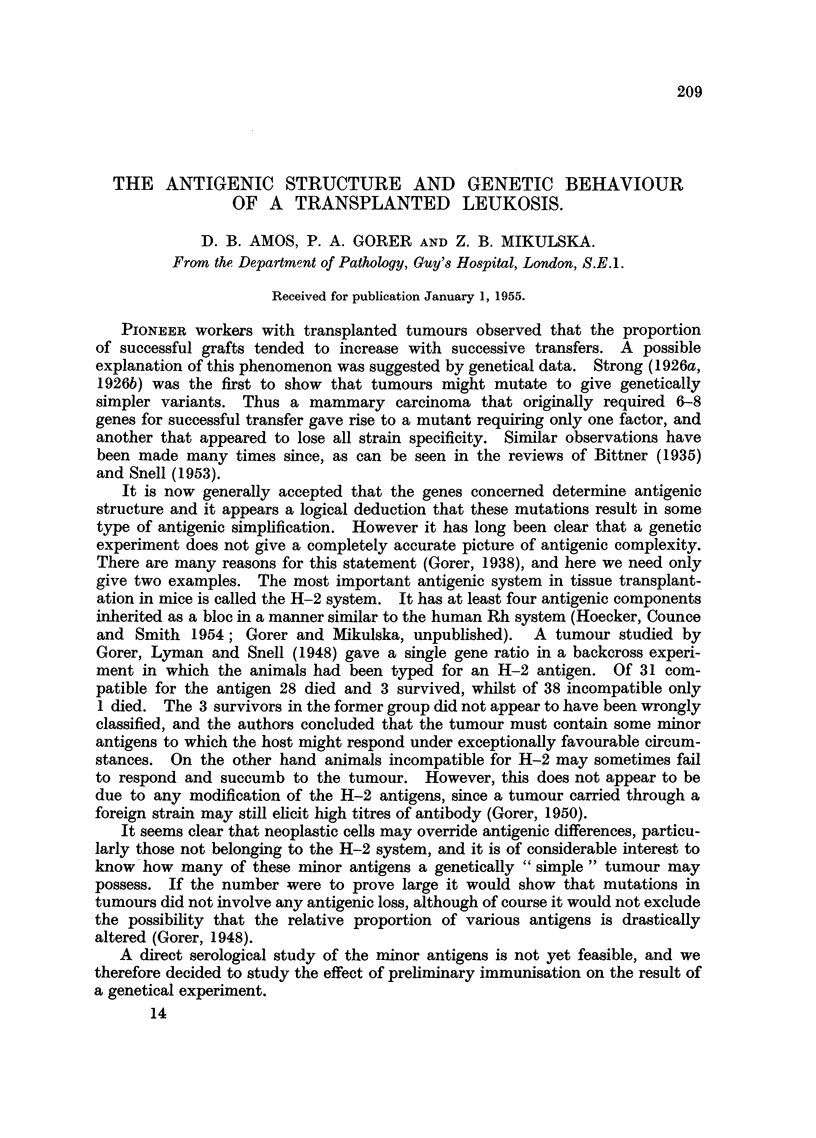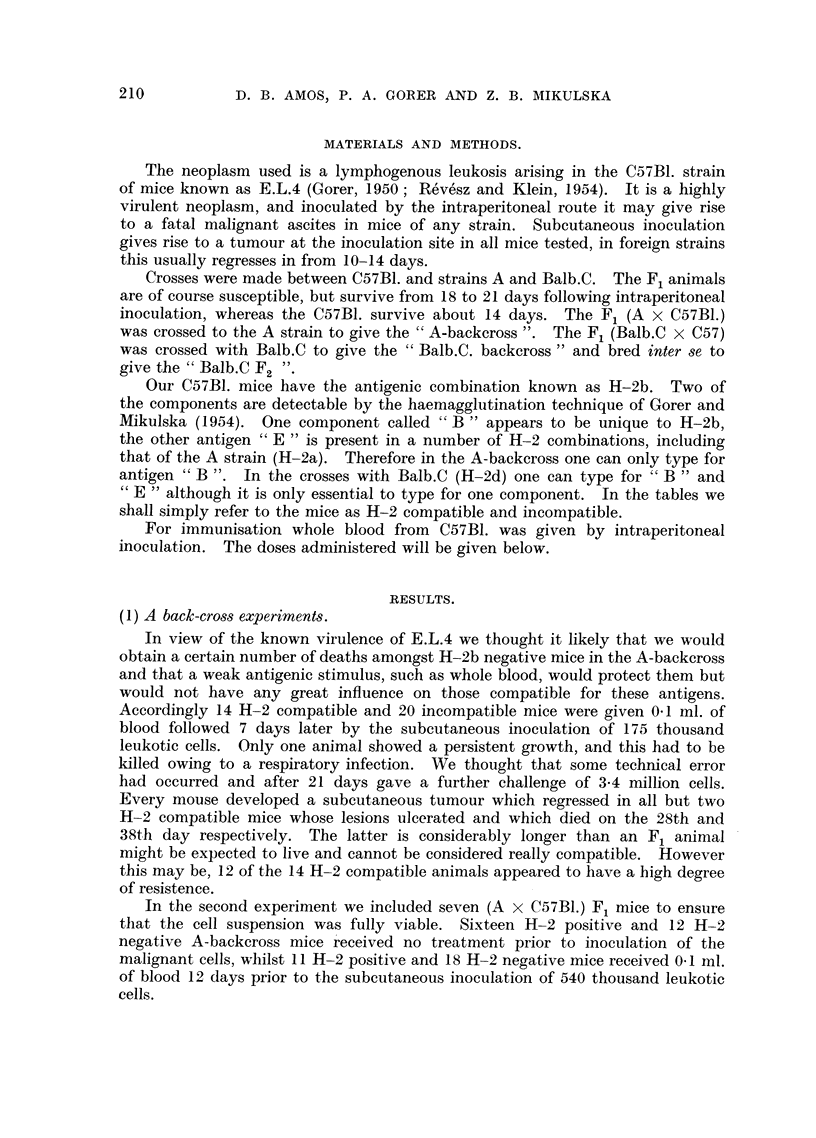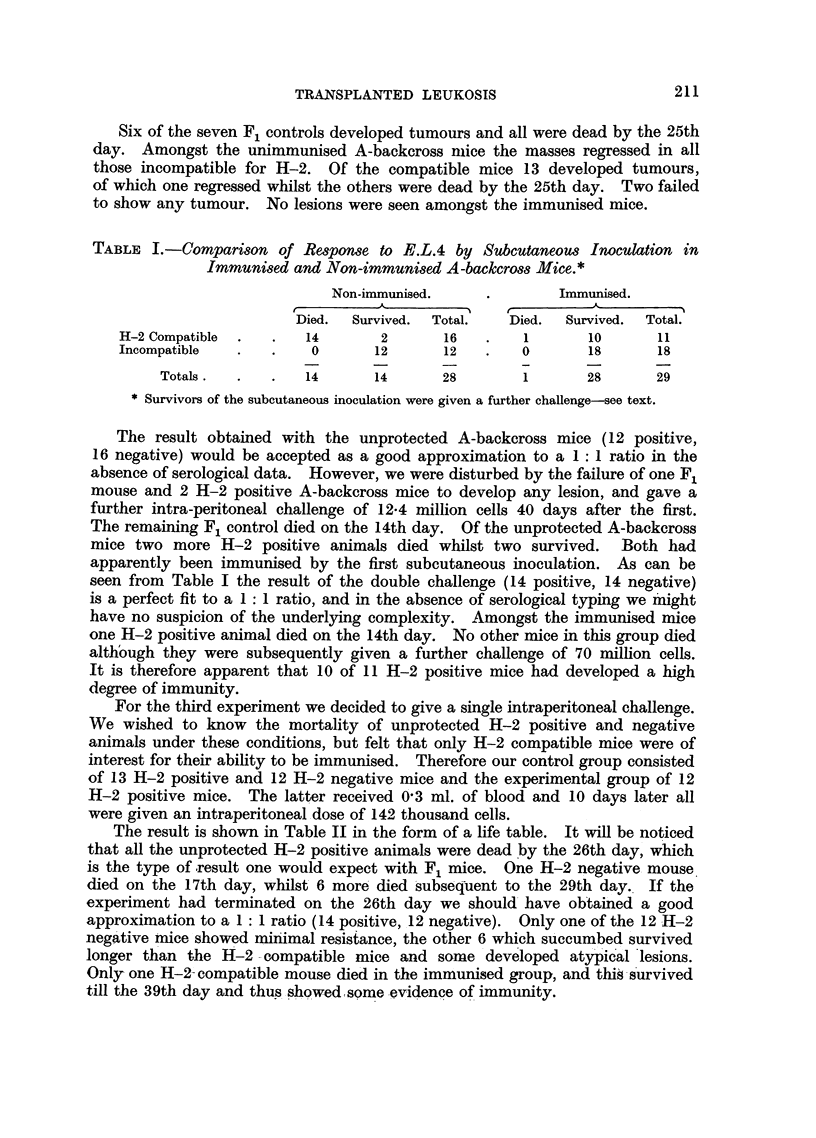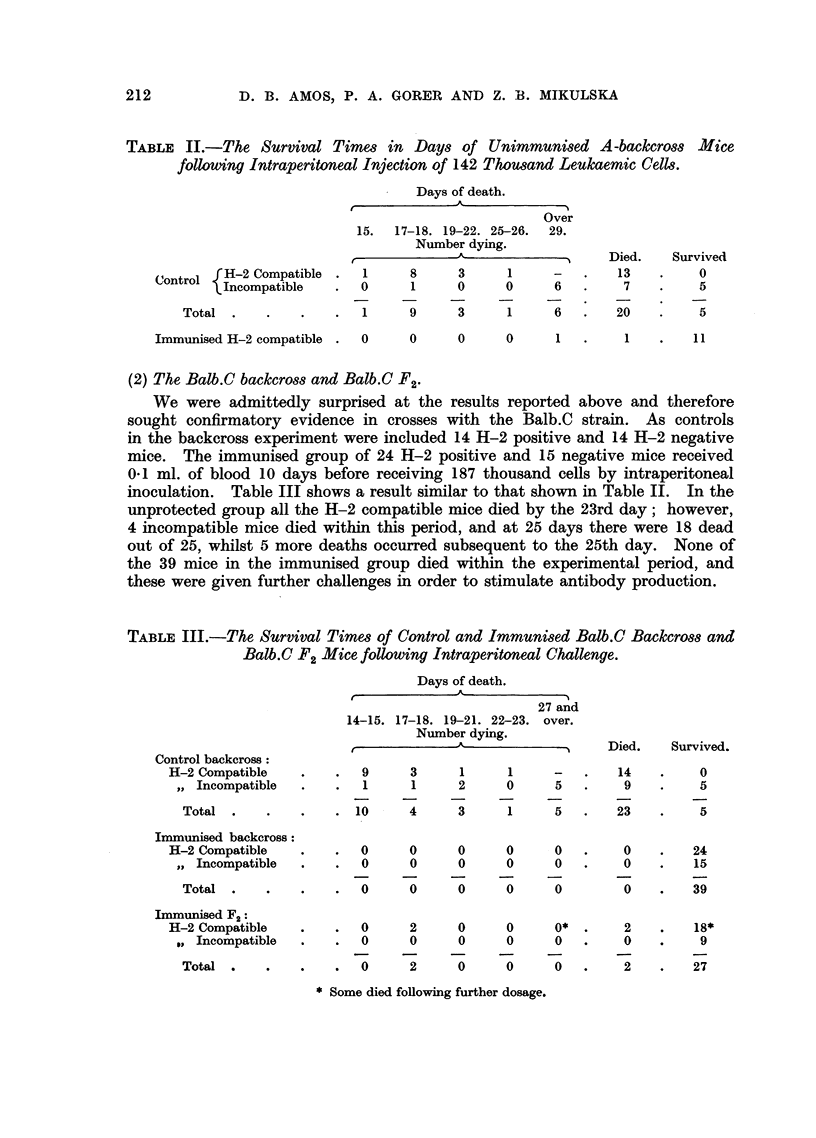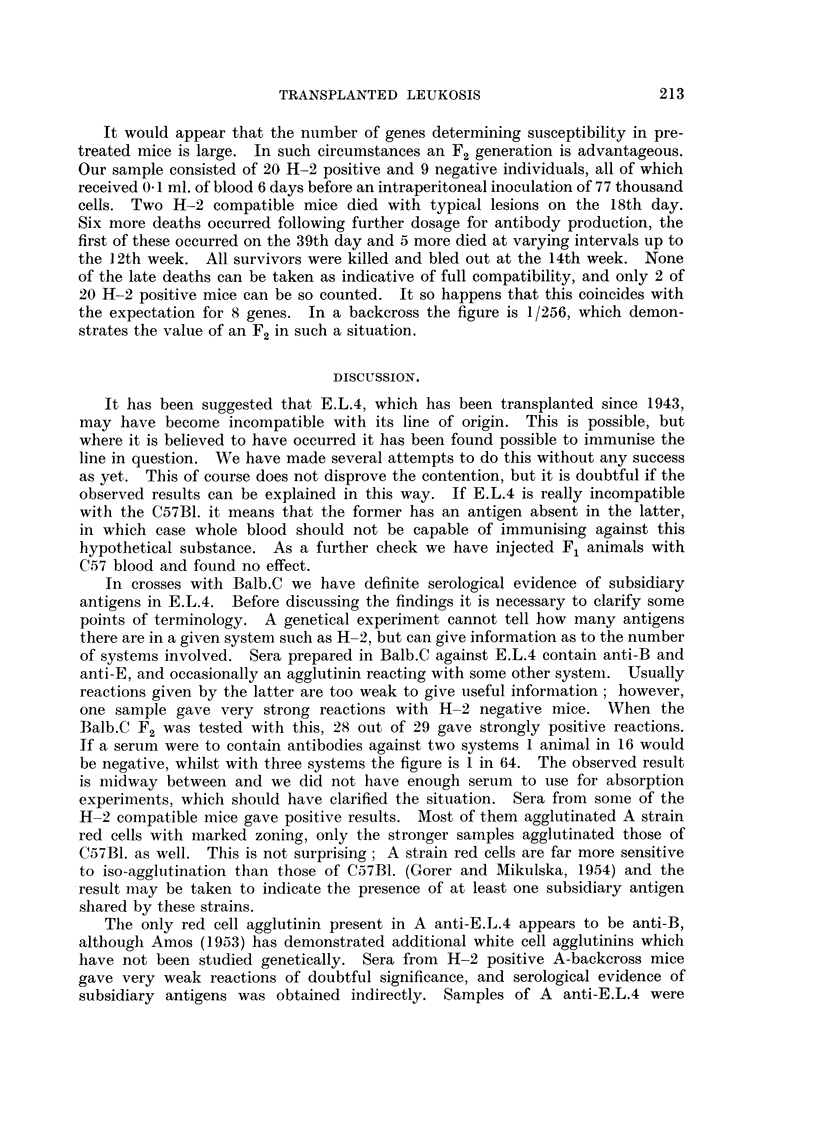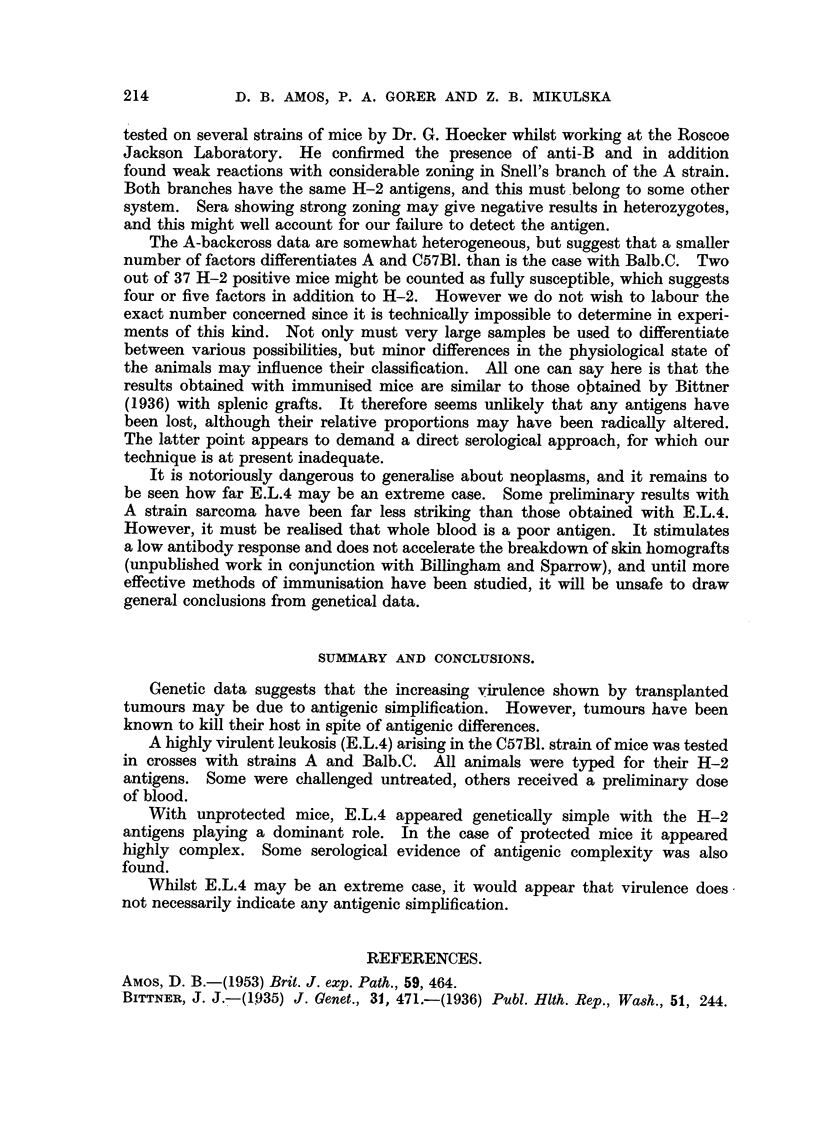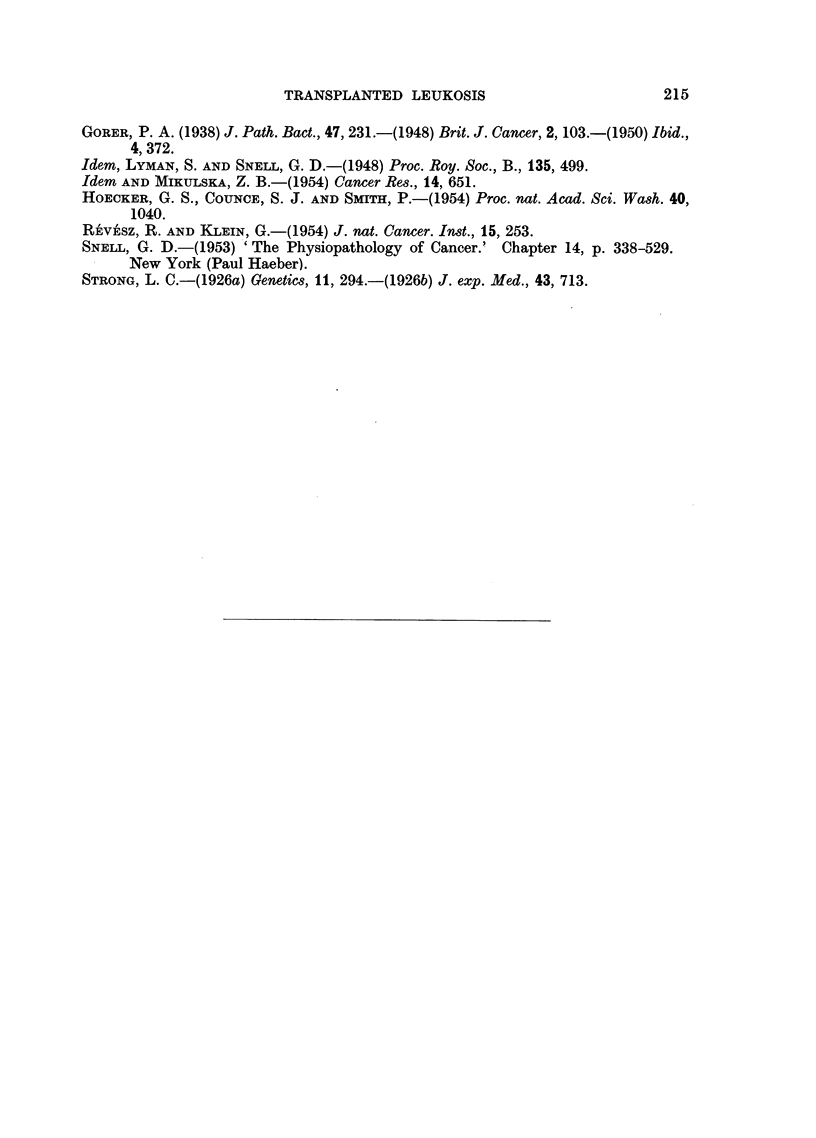# The Antigenic Structure and Genetic Behaviour of a Transplanted Leukosis

**DOI:** 10.1038/bjc.1955.16

**Published:** 1955-03

**Authors:** D. B. Amos, P. A. Gorer, Z. B. Mikulska


### THE ANTIGENIC STRUCTURE AND GENETIC BEHAVIOUR OF A TRANSPLANTED LEUKOSIS.

D. B. AMOS, P. A. GORER AND Z. B. MIKULSKA.

*From the Department of Pathology, Guy's Hospital, London, S.E.*1.

Received for publication January 1, 1955.

PIONEER workers with transplanted tumours observed that the proportion of successful grafts tended to increase with successive transfers. A possible explanation of this phenomenon was suggested by genetical data. Strong (1926*a*, 1926*b*) was the first to show that tumours might mutate to give genetically simpler variants. Thus a mammary carcinoma that originally required 6–8 genes for successful transfer gave rise to a mutant requiring only one factor, and another that appeared to lose all strain specificity. Similar observations have been made many times since, as can be seen in the reviews of Bittner (1935) and Snell (1953).

It is now generally accepted that the genes concerned determine antigenic structure and it appears a logical deduction that these mutations result in some type of antigenic simplification. However it has long been clear that a genetic experiment does not give a completely accurate picture of antigenic complexity. There are many reasons for this statement (Gorer, 1938), and here we need only give two examples. The most important antigenic system in tissue transplantation in mice is called the H–2 system. It has at least four antigenic components inherited as a bloc in a manner similar to the human Rh system (Hoecker, Counce and Smith 1954 ; Gorer and Mikulska, unpublished). A tumour studied by Gorer, Lyman and Snell (1948) gave a single gene ratio in a backcross experiment in which the animals had been typed for an H–2 antigen. Of 31 compatible for the antigen 28 died and 3 survived, whilst of 38 incompatible only 1 died. The 3 survivors in the former group did not appear to have been wrongly classified, and the authors concluded that the tumour must contain some minor antigens to which the host might respond under exceptionally favourable circumstances. On the other hand animals incompatible for H–2 may sometimes fail to respond and succumb to the tumour. However, this does not appear to be due to any modification of the H–2 antigens, since a tumour carried through a foreign strain may still elicit high titres of antibody (Gorer, 1950).

It seems clear that neoplastic cells may override antigenic differences, particularly those not belonging to the H–2 system, and it is of considerable interest to know how many of these minor antigens a genetically " simple " tumour may possess. If the number were to prove large it would show that mutations in tumours did not involve any antigenic loss, although of course it would not exclude the possibility that the relative proportion of various antigens is drastically altered (Gorer, 1948).

A direct serological study of the minor antigens is not yet feasible, and we therefore decided to study the effect of preliminary immunisation on the result of a genetical experiment.

### MATERIALS AND METHODS.

The neoplasm used is a lymphogenous leukosis arising in the C57Bl. strain of mice known as E.L.4 (Gorer, 1950; Révész and Klein, 1954). It is a highly virulent neoplasm, and inoculated by the intraperitoneal route it may give rise to a fatal malignant ascites in mice of any strain. Subcutaneous inoculation gives rise to a tumour at the inoculation site in all mice tested, in foreign strains this usually regresses in from 10–14 days.

Crosses were made between C57Bl. and strains A and Balb.C. The $F_1$ animals are of course susceptible, but survive from 18 to 21 days following intraperitoneal inoculation, whereas the C57Bl. survive about 14 days. The $F_1$ (A $\times$ C57Bl.) was crossed to the A strain to give the " A-backcross ". The $F_1$ (Balb.C $\times$ C57) was crossed with Balb.C to give the " Balb.C. backcross " and bred *inter se* to give the " Balb.C $F_2$ ".

Our C57Bl. mice have the antigenic combination known as H–2b. Two of the components are detectable by the haemagglutination technique of Gorer and Mikulska (1954). One component called " B " appears to be unique to H–2b, the other antigen " E " is present in a number of H–2 combinations, including that of the A strain (H–2a). Therefore in the A-backcross one can only type for antigen " B ". In the crosses with Balb.C (H–2d) one can type for " B " and " E " although it is only essential to type for one component. In the tables we shall simply refer to the mice as H–2 compatible and incompatible.

For immunisation whole blood from C57Bl. was given by intraperitoneal inoculation. The doses administered will be given below.

### RESULTS.

(1) *A back-cross experiments.*

In view of the known virulence of E.L.4 we thought it likely that we would obtain a certain number of deaths amongst H–2b negative mice in the A-backcross and that a weak antigenic stimulus, such as whole blood, would protect them but would not have any great influence on those compatible for these antigens. Accordingly 14 H–2 compatible and 20 incompatible mice were given 0·1 ml. of blood followed 7 days later by the subcutaneous inoculation of 175 thousand leukotic cells. Only one animal showed a persistent growth, and this had to be killed owing to a respiratory infection. We thought that some technical error had occurred and after 21 days gave a further challenge of 3·4 million cells. Every mouse developed a subcutaneous tumour which regressed in all but two H–2 compatible mice whose lesions ulcerated and which died on the 28th and 38th day respectively. The latter is considerably longer than an $F_1$ animal might be expected to live and cannot be considered really compatible. However this may be, 12 of the 14 H–2 compatible animals appeared to have a high degree of resistence.

In the second experiment we included seven (A $\times$ C57Bl.) $F_1$ mice to ensure that the cell suspension was fully viable. Sixteen H–2 positive and 12 H–2 negative A-backcross mice received no treatment prior to inoculation of the malignant cells, whilst 11 H–2 positive and 18 H–2 negative mice received 0·1 ml. of blood 12 days prior to the subcutaneous inoculation of 540 thousand leukotic cells.

Six of the seven $F_1$ controls developed tumours and all were dead by the 25th day. Amongst the unimmunised A-backcross mice the masses regressed in all those incompatible for H–2. Of the compatible mice 13 developed tumours, of which one regressed whilst the others were dead by the 25th day. Two failed to show any tumour. No lesions were seen amongst the immunised mice.

TABLE I.—*Comparison of Response to E.L.4 by Subcutaneous Inoculation in Immunised and Non-immunised A-backcross Mice.**

| | Non-immunised. | | | Immunised. | | |
|---|---|---|---|---|---|---|
| | Died. | Survived. | Total. | Died. | Survived. | Total. |
| H–2 Compatible | 14 | 2 | 16 | 1 | 10 | 11 |
| Incompatible | 0 | 12 | 12 | 0 | 18 | 18 |
| Totals | 14 | 14 | 28 | 1 | 28 | 29 |

* Survivors of the subcutaneous inoculation were given a further challenge—see text.

The result obtained with the unprotected A-backcross mice (12 positive, 16 negative) would be accepted as a good approximation to a 1 : 1 ratio in the absence of serological data. However, we were disturbed by the failure of one $F_1$ mouse and 2 H–2 positive A-backcross mice to develop any lesion, and gave a further intra-peritoneal challenge of 12·4 million cells 40 days after the first. The remaining $F_1$ control died on the 14th day. Of the unprotected A-backcross mice two more H–2 positive animals died whilst two survived. Both had apparently been immunised by the first subcutaneous inoculation. As can be seen from Table I the result of the double challenge (14 positive, 14 negative) is a perfect fit to a 1 : 1 ratio, and in the absence of serological typing we might have no suspicion of the underlying complexity. Amongst the immunised mice one H–2 positive animal died on the 14th day. No other mice in this group died although they were subsequently given a further challenge of 70 million cells. It is therefore apparent that 10 of 11 H–2 positive mice had developed a high degree of immunity.

For the third experiment we decided to give a single intraperitoneal challenge. We wished to know the mortality of unprotected H–2 positive and negative animals under these conditions, but felt that only H–2 compatible mice were of interest for their ability to be immunised. Therefore our control group consisted of 13 H–2 positive and 12 H–2 negative mice and the experimental group of 12 H–2 positive mice. The latter received 0·3 ml. of blood and 10 days later all were given an intraperitoneal dose of 142 thousand cells.

The result is shown in Table II in the form of a life table. It will be noticed that all the unprotected H–2 positive animals were dead by the 26th day, which is the type of result one would expect with $F_1$ mice. One H–2 negative mouse died on the 17th day, whilst 6 more died subsequent to the 29th day. If the experiment had terminated on the 26th day we should have obtained a good approximation to a 1 : 1 ratio (14 positive, 12 negative). Only one of the 12 H–2 negative mice showed minimal resistance, the other 6 which succumbed survived longer than the H–2 compatible mice and some developed atypical lesions. Only one H–2 compatible mouse died in the immunised group, and this survived till the 39th day and thus showed some evidence of immunity.

TABLE II.—*The Survival Times in Days of Unimmunised A-backcross Mice following Intraperitoneal Injection of* 142 *Thousand Leukaemic Cells.*

| | Days of death. 15. | 17–18. | 19–22. | 25–26. | Over 29. | Died. | Survived |
|---|---|---|---|---|---|---|---|
| | Number dying. | | | | | | |
| Control H–2 Compatible | 1 | 8 | 3 | 1 | – | 13 | 0 |
| Control Incompatible | 0 | 1 | 0 | 0 | 6 | 7 | 5 |
| Total | 1 | 9 | 3 | 1 | 6 | 20 | 5 |
| Immunised H–2 compatible | 0 | 0 | 0 | 0 | 1 | 1 | 11 |

(2) *The Balb.C backcross and Balb.C* $F_2$.

We were admittedly surprised at the results reported above and therefore sought confirmatory evidence in crosses with the Balb.C strain. As controls in the backcross experiment were included 14 H–2 positive and 14 H–2 negative mice. The immunised group of 24 H–2 positive and 15 negative mice received 0·1 ml. of blood 10 days before receiving 187 thousand cells by intraperitoneal inoculation. Table III shows a result similar to that shown in Table II. In the unprotected group all the H–2 compatible mice died by the 23rd day ; however, 4 incompatible mice died within this period, and at 25 days there were 18 dead out of 25, whilst 5 more deaths occurred subsequent to the 25th day. None of the 39 mice in the immunised group died within the experimental period, and these were given further challenges in order to stimulate antibody production.

TABLE III.—*The Survival Times of Control and Immunised Balb.C Backcross and Balb.C* $F_2$ *Mice following Intraperitoneal Challenge.*

| | Days of death. 14–15. | 17–18. | 19–21. | 22–23. | 27 and over. | Died. | Survived. |
|---|---|---|---|---|---|---|---|
| | Number dying. | | | | | | |
| Control backcross : | | | | | | | |
| H–2 Compatible | 9 | 3 | 1 | 1 | – | 14 | 0 |
| „ Incompatible | 1 | 1 | 2 | 0 | 5 | 9 | 5 |
| Total | 10 | 4 | 3 | 1 | 5 | 23 | 5 |
| Immunised backcross : | | | | | | | |
| H–2 Compatible | 0 | 0 | 0 | 0 | 0 | 0 | 24 |
| „ Incompatible | 0 | 0 | 0 | 0 | 0 | 0 | 15 |
| Total | 0 | 0 | 0 | 0 | 0 | 0 | 39 |
| Immunised $F_2$ : | | | | | | | |
| H–2 Compatible | 0 | 2 | 0 | 0 | 0* | 2 | 18* |
| „ Incompatible | 0 | 0 | 0 | 0 | 0 | 0 | 9 |
| Total | 0 | 2 | 0 | 0 | 0 | 2 | 27 |

* Some died following further dosage.

It would appear that the number of genes determining susceptibility in pretreated mice is large. In such circumstances an $F_2$ generation is advantageous. Our sample consisted of 20 H–2 positive and 9 negative individuals, all of which received 0·1 ml. of blood 6 days before an intraperitoneal inoculation of 77 thousand cells. Two H–2 compatible mice died with typical lesions on the 18th day. Six more deaths occurred following further dosage for antibody production, the first of these occurred on the 39th day and 5 more died at varying intervals up to the 12th week. All survivors were killed and bled out at the 14th week. None of the late deaths can be taken as indicative of full compatibility, and only 2 of 20 H–2 positive mice can be so counted. It so happens that this coincides with the expectation for 8 genes. In a backcross the figure is 1/256, which demonstrates the value of an $F_2$ in such a situation.

### DISCUSSION.

It has been suggested that E.L.4, which has been transplanted since 1943, may have become incompatible with its line of origin. This is possible, but where it is believed to have occurred it has been found possible to immunise the line in question. We have made several attempts to do this without any success as yet. This of course does not disprove the contention, but it is doubtful if the observed results can be explained in this way. If E.L.4 is really incompatible with the C57Bl. it means that the former has an antigen absent in the latter, in which case whole blood should not be capable of immunising against this hypothetical substance. As a further check we have injected $F_1$ animals with C57 blood and found no effect.

In crosses with Balb.C we have definite serological evidence of subsidiary antigens in E.L.4. Before discussing the findings it is necessary to clarify some points of terminology. A genetical experiment cannot tell how many antigens there are in a given system such as H–2, but can give information as to the number of systems involved. Sera prepared in Balb.C against E.L.4 contain anti-B and anti-E, and occasionally an agglutinin reacting with some other system. Usually reactions given by the latter are too weak to give useful information ; however, one sample gave very strong reactions with H–2 negative mice. When the Balb.C $F_2$ was tested with this, 28 out of 29 gave strongly positive reactions. If a serum were to contain antibodies against two systems 1 animal in 16 would be negative, whilst with three systems the figure is 1 in 64. The observed result is midway between and we did not have enough serum to use for absorption experiments, which should have clarified the situation. Sera from some of the H–2 compatible mice gave positive results. Most of them agglutinated A strain red cells with marked zoning, only the stronger samples agglutinated those of C57Bl. as well. This is not surprising ; A strain red cells are far more sensitive to iso-agglutination than those of C57Bl. (Gorer and Mikulska, 1954) and the result may be taken to indicate the presence of at least one subsidiary antigen shared by these strains.

The only red cell agglutinin present in A anti-E.L.4 appears to be anti-B, although Amos (1953) has demonstrated additional white cell agglutinins which have not been studied genetically. Sera from H–2 positive A-backcross mice gave very weak reactions of doubtful significance, and serological evidence of subsidiary antigens was obtained indirectly. Samples of A anti-E.L.4 were tested on several strains of mice by Dr. G. Hoecker whilst working at the Roscoe Jackson Laboratory. He confirmed the presence of anti-B and in addition found weak reactions with considerable zoning in Snell's branch of the A strain. Both branches have the same H–2 antigens, and this must belong to some other system. Sera showing strong zoning may give negative results in heterozygotes, and this might well account for our failure to detect the antigen.

The A-backcross data are somewhat heterogeneous, but suggest that a smaller number of factors differentiates A and C57Bl. than is the case with Balb.C. Two out of 37 H–2 positive mice might be counted as fully susceptible, which suggests four or five factors in addition to H–2. However we do not wish to labour the exact number concerned since it is technically impossible to determine in experiments of this kind. Not only must very large samples be used to differentiate between various possibilities, but minor differences in the physiological state of the animals may influence their classification. All one can say here is that the results obtained with immunised mice are similar to those obtained by Bittner (1936) with splenic grafts. It therefore seems unlikely that any antigens have been lost, although their relative proportions may have been radically altered. The latter point appears to demand a direct serological approach, for which our technique is at present inadequate.

It is notoriously dangerous to generalise about neoplasms, and it remains to be seen how far E.L.4 may be an extreme case. Some preliminary results with A strain sarcoma have been far less striking than those obtained with E.L.4. However, it must be realised that whole blood is a poor antigen. It stimulates a low antibody response and does not accelerate the breakdown of skin homografts (unpublished work in conjunction with Billingham and Sparrow), and until more effective methods of immunisation have been studied, it will be unsafe to draw general conclusions from genetical data.

### SUMMARY AND CONCLUSIONS.

Genetic data suggests that the increasing virulence shown by transplanted tumours may be due to antigenic simplification. However, tumours have been known to kill their host in spite of antigenic differences.

A highly virulent leukosis (E.L.4) arising in the C57Bl. strain of mice was tested in crosses with strains A and Balb.C. All animals were typed for their H–2 antigens. Some were challenged untreated, others received a preliminary dose of blood.

With unprotected mice, E.L.4 appeared genetically simple with the H–2 antigens playing a dominant role. In the case of protected mice it appeared highly complex. Some serological evidence of antigenic complexity was also found.

Whilst E.L.4 may be an extreme case, it would appear that virulence does not necessarily indicate any antigenic simplification.

### REFERENCES.

AMOS, D. B.—(1953) *Brit. J. exp. Path.*, **59**, 464.
BITTNER, J. J.—(1935) *J. Genet.*, **31**, 471.—(1936) *Publ. Hlth. Rep., Wash.*, **51**, 244.

GORER, P. A. (1938) *J. Path. Bact.*, **47**, 231.—(1948) *Brit. J. Cancer*, **2**, 103.—(1950) *Ibid.*, **4**, 372.
*Idem*, LYMAN, S. AND SNELL, G. D.—(1948) *Proc. Roy. Soc.*, B., **135**, 499.
*Idem* AND MIKULSKA, Z. B.—(1954) *Cancer Res.*, **14**, 651.
HOECKER, G. S., COUNCE, S. J. AND SMITH, P.—(1954) *Proc. nat. Acad. Sci. Wash.* **40**, 1040.
RÉVÉSZ, R. AND KLEIN, G.—(1954) *J. nat. Cancer. Inst.*, **15**, 253.
SNELL, G. D.—(1953) 'The Physiopathology of Cancer.' Chapter 14, p. 338–529. New York (Paul Haeber).
STRONG, L. C.—(1926*a*) *Genetics*, **11**, 294.—(1926*b*) *J. exp. Med.*, **43**, 713.